# Pullulan/dextran/nHA Macroporous Composite Beads for Bone Repair in a Femoral Condyle Defect in Rats

**DOI:** 10.1371/journal.pone.0110251

**Published:** 2014-10-20

**Authors:** Silke Schlaubitz, Sidi Mohammed Derkaoui, Lydia Marosa, Sylvain Miraux, Martine Renard, Sylvain Catros, Catherine Le Visage, Didier Letourneur, Joëlle Amédée, Jean-Christophe Fricain

**Affiliations:** 1 CIC 1401, University hospital of Bordeaux/Inserm, Bordeaux, France; 2 U1148, LVTS/Inserm, Paris, France; 3 Près Sorbonne Paris Cité, University of Paris Nord and University Paris Diderot, Paris, France; 4 U1026 Tissue Bioengineering, University of Bordeaux/Inserm, Bordeaux, France; 5 RMSB Center/UMR 5536, CNRS, Bordeaux, France; 6 Dental School, University of Bordeaux, Bordeaux, France; Faculté de médecine de Nantes, France

## Abstract

The repair of bone defects is of particular interest for orthopedic, oral, maxillofacial, and dental surgery. Bone loss requiring reconstruction is conventionally addressed through bone grafting. Depending on the size and the location of the defect, this method has limits and risks. Biomaterials can offer an alternative and have features supporting bone repair. Here, we propose to evaluate the cellular penetration and bone formation of new macroporous beads based on pullulan/dextran that has been supplemented with nanocrystalline hydroxyapatite in a rat model. Cross-linked beads of 300–500 µm diameters were used in a lateral femoral condyle defect and analyzed by magnetic resonance imaging, micro-computed tomography, and histology in comparison to the empty defects 15, 30, and 70 days after implantation. Inflammation was absent for both conditions. For empty defects, cellularisation and mineralization started from the periphery of the defect. For the defects containing beads, cellular structures filling out the spaces between the scaffolds with increasing interconnectivity and trabecular-like organization were observed over time. The analysis of calcified sections showed increased mineralization over time for both conditions, but was more pronounced for the samples containing beads. Bone Mineral Density and Bone Mineral Content were both significantly higher at day 70 for the beads in comparison to empty defects as well as compared with earlier time points. Analysis of newly formed tissue around the beads showed an increase of osteoid tissue, measured as percentage of the defect surface. This study suggests that the use of beads for the repair of small size defects in bone may be expanded on to meet the clinical need for a ready-to-use fill-up material that can favor bone formation and mineralization, as well as promote vessel ingrowth into the defect site.

## Introduction

The repair of bone defects caused by trauma, surgery or due to cancer treatment is of particular interest for surgeons. Conventional bone grafting is still the gold standard, but depending on the size and the location of the defect, this method has limits and risks regarding the efficiency, possible infections and the manageable pain [1]. Biomaterials can offer an alternative and have osteoconductive [2], [3] and/or osteoinductive [4]–[6] properties supporting bone repair while being biocompatible and biodegradable [7]–[12].

Bone substitute biomaterials are often based on calcium phosphates like hydroxyapatite (HA) [13], tricalcium phosphate (TCP) [13]–[15], biphasic calcium phosphates (BCP) [13], [16], [17], or coral [18] because of their biocompatibility due to a composition similar to mature bone, and their osteoconductive properties [2]. The use of calcium phosphate itself is limited to small defects, where biomaterials with osteoconductive properties are sufficient for bone reconstruction [5], or as a coating of other biomaterials [19], because those materials allow only limited neo-angiogenesis and are therefore not suitable for larger size defects.

Other groups of bone substitute biomaterials that promote vessel ingrowth are mainly based on polymers. The properties of natural polymers like collagen [20], [21], dextran [22], chitosan [23], alginate/fibrin [24] or synthetic polymers like polyglycolic acid (PGA), polylactic acid (PLA), and poly(lactic-*co*-glycolic) acid (PLGA) among others [25], [26] have been extensively studied in *in* *vitro* bone tissue engineering applications, mainly as delivery systems for encapsulated stem cells or growth factors. Polymer scaffolds can easily be generated in various 3D structures such as macroporous scaffolds, biospun scaffolds, micro beads, hydrogels, micro-molded matrices and nanoparticles [27]. Some polymers have little affinity with bone, but can easily be associated with cells or growth factors to promote bone tissue regeneration with angiogenic potential *in* *vivo* [28]–[32]. To combine properties favoring bone affinity and angiogenesis, specific composites of polymers and ceramics are being developed that have shown osteoinductive and osteoconductive properties [33]. Composite materials in different shape and sizes of scaffolds have been used in numerous *in* *vivo* investigations to promote bone healing [34]–[39], and can easily be associated with cells or growth factors to enhance osteoinductive and/or osteoconductive properties [40], [41]. However the use of composite beads seems preferable for some clinical applications, because they could be injectable and therefore easy to handle and adapt to the morphology of any defect [42], as seen previously in a clinical trial [43].

Injectable beads have been studied *in* *vitro* as composite materials such as alginate/β-tricalcium phosphate [44], Chitosan-collagen/nano-hydroxyapatite [45], or fibrin/β-tricalcium phosphate [46], but *in* *vivo* studies have not been performed with those materials yet.

In the present study, a biomaterial based on a mixture of pullulan and dextran in association with nanocrystalline hydroxyapatite designed as cross-linked micro-beads was used as a scaffold. Previous experiments have shown the osteoconductive properties of the biomaterial when used as a massive macroporous scaffold [36]. In this study, the objective was to demonstrate for the first time that beads of a new ready-to-use fill-up composite material made of dextran/pullulan/nanocrystalline hydroxyapatite are suitable materials to activate and promote bone regeneration and mineralization in rat femoral condyle defects.

## Materials and Methods

### Preparation and characterization of the biomaterial

A nanocrystalline hydroxyapatite (nHA) solution was prepared by wet chemical precipitation using a 0.6 M solution of phosphoric acid (Prolabo, Paris, France) and a 1 M solution of calcium hydroxide (Alfa Aesar, Germany) [47], [48]. The nHA powder was composed of 50–100 nm long rod-like crystals as observed by transmission electron microscopy. X ray quantitative micro analysis revealed a Ca/P ratio of 1.67 and FITR analysis confirmed the presence of phosphate, hydroxide and carbonate groups, as described previously [47].

Nanocrystalline hydroxyapatite-polysaccharide microparticles composite were obtained using water-in-oil (w/o) emulsification process. Briefly, 75% pullulan (MW 200000, Hayashibara Inc., Japan) and 25% dextran (MW 500000, Sigma, France) were dissolved in nHA suspension and NaCl as a porogen. The mixture was then dispersed in canola oil under mechanical stirring and polysaccharide beads were cross-linked by sodium trimetaphosphate at 50°C for 20 min. Resulting microbeads were extensively washed with 0.025% NaCl solution, and sieved, using a vibrating shaker (AS 200, Retsch, France) to obtain beads of 300–500 µm diameter that were freeze-dried [49].

Hydrated scaffolds were observed using environmental scanning electron microscopy (ESEM) with a Philips XL 30 ESEM-FEG (Eindhoven, The Netherlands) at an accelerating voltage of 15 keV and at a pressure of 3.5 Torr. Dry beads were observed by Back Scattered Electron Microscopy (BSEM) after gold sputtering on a 6700F electron microscope (JEOL, Peabody/MA, USA) working at 10 keV.

### *In vivo* experiments

Animal experiments were performed in accordance with the “Principles of Laboratory Animal Care” recommended by the National Society for Biomedical Research in France. Interventions were carried out in an accredited animal facility (authorization n° A33-063-917) at the University of Bordeaux, under authorization n° B3310023 of the French Ministry of Agriculture and were approved by the Animal Ethic Committee of the University of Bordeaux.

Anesthesia was induced with a mixture of 4% isoflurane/Air 1–2 L/min (Baxter, Deerfield/IL, USA) and was maintained at 2% isoflurane/Air 0.8 L/min during the implantation. A stable body temperature was assured by the use of a heating device.

Medial defects of 3.5 mm diameter and 4 mm depth were introduced in left and right femoral condyles of 18 female Wistar RjHan rats at 10 weeks of age weighting 250–300 g (Janvier, Le Genest, France) using a dental microdrill (Thomas, France). Bone defects of about 38 mm^3^ were rinsed with physiological solution NaCl 0.9% (w/v). The defect was either left empty or filled with 17±3 dry beads that were implanted into the site, leaving enough time for the beads to hydrate with the blood invading the defect and, as a consequence, to increase slightly in size. The scaffolds remained solid enough to stay inside the defect. Therefore, no membrane was needed to avoid leakage of the biomaterial. Absorbable Vicryl sutures were performed on lateral muscles before closing the cutaneous plan with non-absorbable prolene sutures and suture clips.

To assure analgesia, 0.3 mg/kg of buprenorphine (Vetergesic Multidose, Alstoe Veterinary, York, UK) was administered subcutaneously to each rat 30 min prior to the procedure as well as 24 hours afterwards. Additionally, animals were kept under free access to 0.2 mg/mL of Ibuprofen (Advil, Pfizer, Canada) 3 days prior to surgery and during 1 week after surgery through the drinking water. Twelve animals were euthanized by an overdose of CO_2_ and the six animals that were followed longitudinally by MRI and therefore housed in a different facility, were euthanized by lethal injection (200 mg/animal) of sodium pentobarbital (Ceva, France) for the last time point when the regulator delivering the CO_2_ was not available. The implants and the surrounding tissues were recovered 15, 30 and 70 days after surgery and fixed in 4% (w/v) paraformaldehyde for 4 days at 4°C. After rinsing in 0.1 M phosphate buffer pH 7.4, samples were processed for Micro-CT analysis and histology. For each time point, 6 condyles were left empty and 6 condyles were filled with the beads as matrices.

### Magnetic Resonance Imaging

Immune response and degradation of the implanted scaffold was followed longitudinally in 6 rats by Magnetic Resonance Imaging (MRI). The rats were anaesthetized with 1–1.5% isoflurane/Air 0.8 L/min and maintained at a constant respiration rate of 75±15 respirations/min. Acquisitions at day 15, 30, and 70 after implantation were obtained with a 4.7T Biospec system (Bruker, Ettlingen, Germany) equipped with a 12 cm gradient system capable of 200 mT/m maximum strength. Measurements were performed with a Helmoltz coil (35/25 mm diameter) tuned to 200.3 MHz.

The animals were put in the prone position within the magnet, with the knee at the center of the NMR coil. A 3D TrueFISP imaging with alternating RF phase pulse method and sum of square reconstruction was used as described previously [50]. The aqueous volume was measured for the defect of each acquisition and the results are presented as the means of 6 samples with standard deviation.

### X-ray Micro-Computed Tomography (Micro-CT)

Three-dimensional images of the implants and surrounding tissue were produced by Micro-CT and used for quantification of the newly formed bone. *Ex vivo* acquisitions at 15 µm resolution were performed from 900 X-ray radiographs obtained on an Explore Locus SP X-Ray µCT device (General Electric, Milwaukee/WI, USA) with a source voltage of 80 kV, a current of 80 µA, and an exposure time of 3000 ms. Reconstruction of the region of interest was performed after correction of the center of rotation and calibration of mineral density. After calibration, three dimensional analyses were performed using Microview software (*GE Healthcare* Inc., Princeton/NJ, USA). The threshold for newly formed bone was determined for each scan individually. Quantification included Bone Mineral density (BMD), and Bone Mineral Content (BMC) as defined previously [51]. BMC values were analyzed from regions of interest (ROI) of a fixed volume. Six ROI throughout the defect were determined for each sample. The means of all ROI per sample were analyzed further to evaluate the means with standard deviation of six samples per condition at each time point. For the analysis of BMD, the entire defect site of six samples per condition was taken into account and the results of six samples per condition are shown as the means with standard deviation.

### Histology

Each sample was cut median between the two femoral condyles. One half of each sample was decalcified for 7 hours in a commercially available decalcification solution (DC3, Labonord, France), dehydrated and embedded in paraffin, while the other half was processed for resin inclusion in serial changes of 5% (w/v) Methyl benzoate (Sigma Aldrich), 35% (w/v) Butyl methacrylate (Sigma Aldrich), and 60% (w/v) Methyl methacrylate (VWR, Radnor/PA, USA) with increasing amounts of 4,N,N-Trimethylaniline (Sigma Aldrich, St. Louis/MO, USA).

Sections of 4–10 µm were obtained from each sample and stained with Masson’s Trichrome (decalcified samples) or Von Kossa (calcified samples). Pictures were analyzed with an Eclipse 80i microscope light microscopy (Nikon, Japan) and captured using a DXM 1200C CCD camera (Nikon, Japan).

### Immunohistochemistry

Infiltration of macrophages was evaluated by immunohistochemistry for transmembrane glycoprotein CD68, belonging to the lysosomal-associated membrane protein family. Vessel formation was investigated for the presence of the angiogenesis marker von Willebrand Factor, and the presence of osteoblast precursors was evaluated with immune-staining for BMP-2. Sections were deparaffinized in two changes of toluene for 5 min. Endogenous peroxidase was blocked by applying 3% (v/v) hydrogen peroxide in methanol for 10 min at −20°C. Antigen Retrieval was performed by applying Proteinase K (Invitrogen, Grand Island/NY, USA) for 10 min at room temperature. Endogenous biotin was blocked using an avidin/biotin blocking kit (Invitrogen, Grand Island/NY, USA), before blocking unspecific antibody binding using Histostain-SP kit (Invitrogen, Grand Island/NY, USA). Mouse monoclonal anti-rat Cluster of Differentiation 68 (CD68) antibody (AbD Serotec MCA341GA, Kidlington, UK; dilution 1∶50), rabbit polyclonal anti rat Bone Morphogenetic Protein 2 (BMP2) antibody (Abcam 14933, Cambridge/MA, USA; dilution 1∶250), and mouse monoclonal anti-rat von Willebrand Factor (vWF) antibody (DAKO A0082, Carpinteria/CA, USA; dilution 1∶200) were all applied for 1 hour at room temperature. A biotinylated secondary antibody and the enzyme conjugate were applied for 10 minutes each, followed by 3 min with the AEC chromogen substrate (Histostain-SP kit, Invitrogen, Grand Island/NY, USA). Counterstaining was performed for 1 min with Mayer’s hemalun (VWR, Radnor/PA, USA). Immune-staining was evaluated with an Eclipse 80i microscope light microscopy (Nikon, Tokyo, Japan).

### Histomorphometry

The percentage of newly formed osteoid tissue in defects filled with beads was determined from histological paraffin sections using NIS-Elements software (Nikon, Japan). Osteoid formation that was stained in dark red by Trichrome’s Masson staining protocol using 2 volumes of 1% (v/v) Fushine acid and 3 volumes of 1% (v/v) Xylidine Ponceau, was detected semi-automatically by the software. For samples that were left empty, the surface of osteoid was expressed as a percentage of defect surface. For samples that received the beads, two analyses were performed. Once, we performed the same analysis and once we took into account the invading tissue only, subtracting the surface of the beads from the defect surface. Histologically stained slides from three samples per condition were processed for analysis, and 12 to 19 sections were analyzed per condition. Results are shown as means with standard deviation per condition.

### Statistical analysis

Statistical significance was evaluated with a non-parametric “One-way Anova analysis of variance” followed by a Tukey’s Multiple Comparison Test to compare all possible pairs of means independently, as provided by the GraphPad Prism Software (La Jolla/CA, USA). The significance level was set to alpha equal 0.05. Statistical significances are marked by stars with * indicating *p*≤0.05, ** *p*≤0.01, and *** *p*≤0.001.

## Results

**Figure 1:**
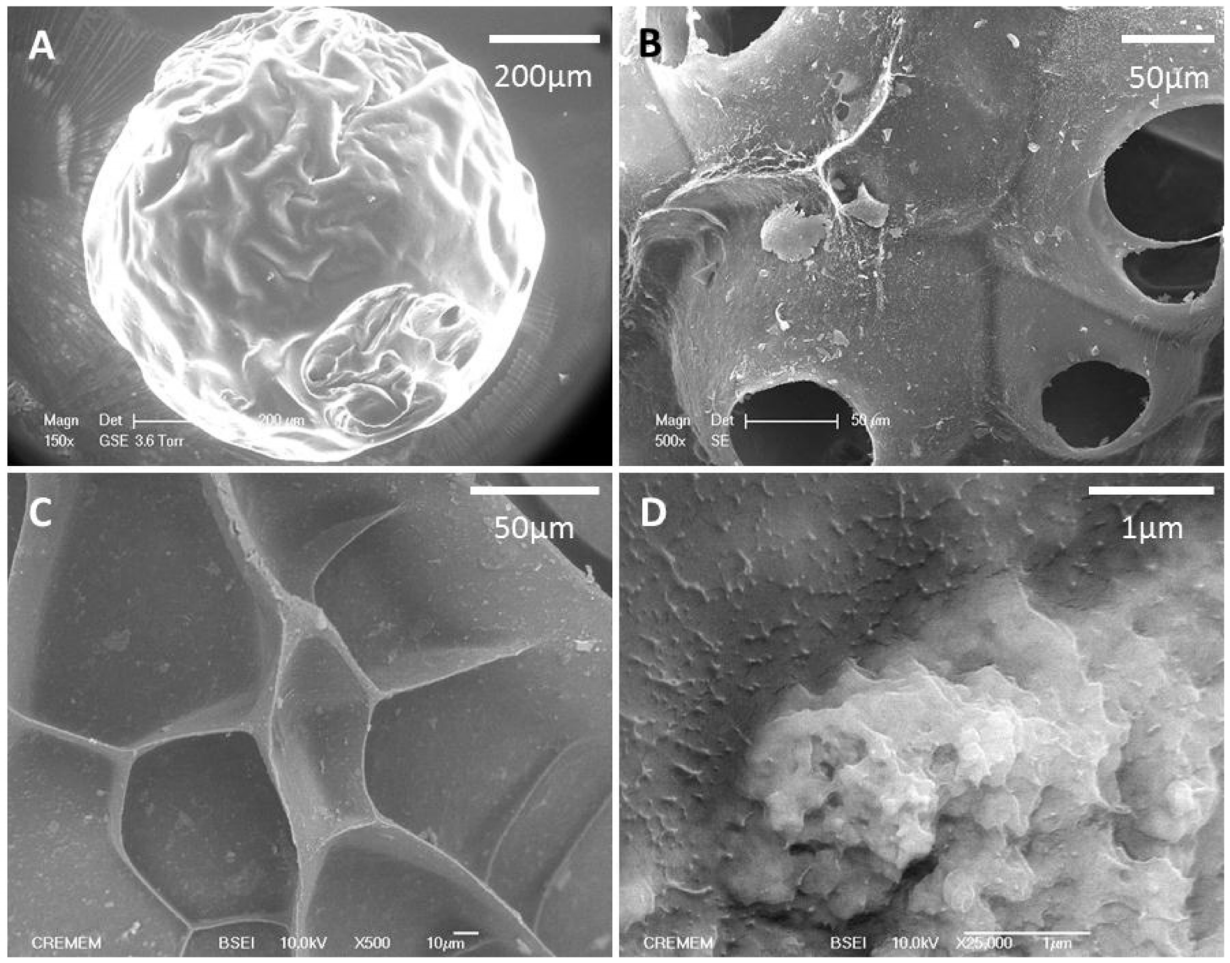
The morphology of microspheres was analyzed by scanning electron microscopy (A). After rehydration in PBS, porosity of hydrated scaffolds was observed with an Environmental Scanning Electron Microscopy (B). Back-scattered Electron Microscopy images of dry beads show pores and overall surface properties (C) and the distribution of nHA aggregates within the structure (D).

Chemical cross-linking with sodium trimetaphosphate of an alkaline solution containing NaCl as a porogen, nHA and a polysaccharide blend dispersed into canola oil under mechanical stirring allowed obtaining the beads. With this patented emulsification process [49], the resulting water-insoluble spherical beads were calibrated according to their size and then freeze-dried. The beads contained 2.8±0.1% (w/v) of nanocrystalline hydroxyapatite. Beads of 300–500 µm in diameter (Figure 1A) were observed by Back-scattered Electron Microscopy (BSEM), revealing micropores and a distribution of the nHA particles within the structure (Figure 1B–D).

**Figure 2:**
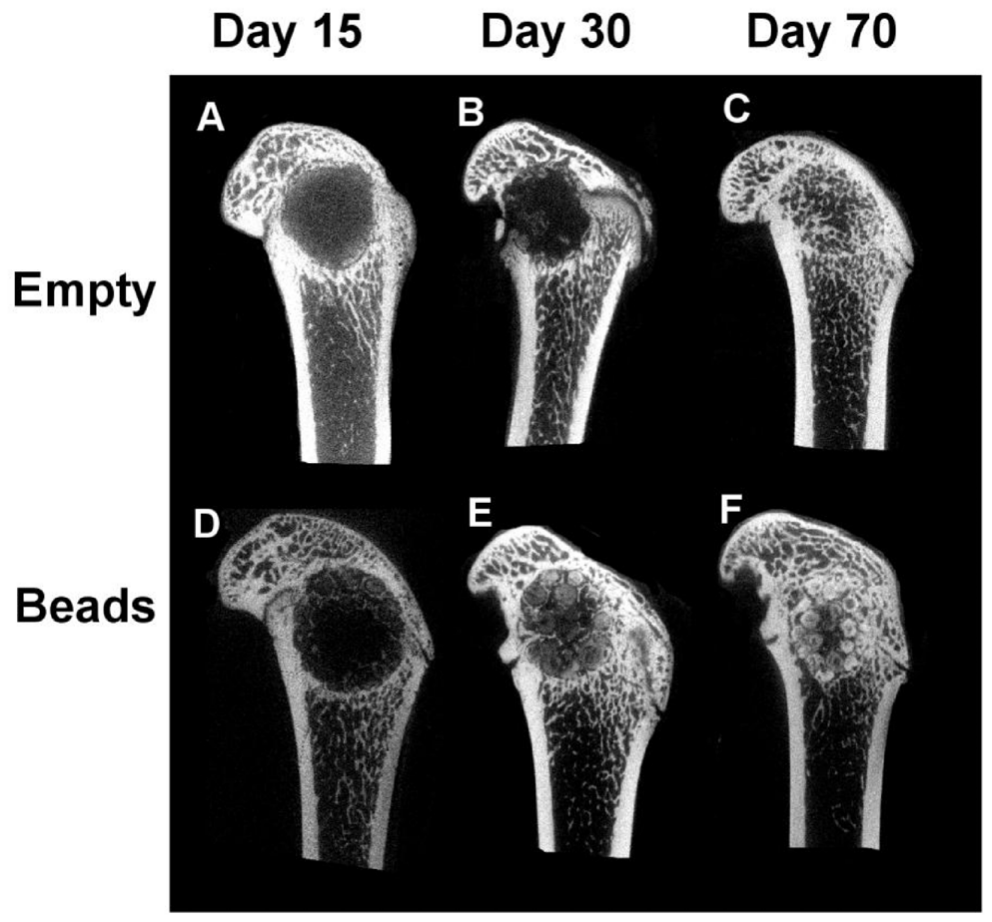
Representative 2D pictures of the median region of Micro-CT 3D reconstructed femoral condyles that were left empty (A–C) or received beads as implants (D–F). Mineralization around the beads is visible as early as 15 days after surgery (D). Empty defects show little mineralization within the defect 30 days after implantation (B), starting from the periphery of the defects. At day 70, mineralization is visible within the defect site for both conditions (C, F).

2D images of 3D Micro-CT reconstructed femoral condyles after implantation of the beads in comparison with defects left empty revealed that mineralization occurred in both groups, empty defect and defect filled with beads. In the median of the defect, there was an increase in mineralized tissue over time of implantation (day 15 through day 70) (Figure 2) that seemed more pronounced and occurred faster for samples that received beads (Figure 2 D–F) than for defects that were left empty (Figure 2 A–C). At day 15, fine trabecular-like structures were only visible around the beads. These trabecular-like structures became more sophisticated over time. Empty defects showed little mineralization in the median of the defect after 30 days after implantation, starting from the periphery of the defects. At day 70, mineralization in form of trabecular structures was visible within the defect site.

**Figure 3:**
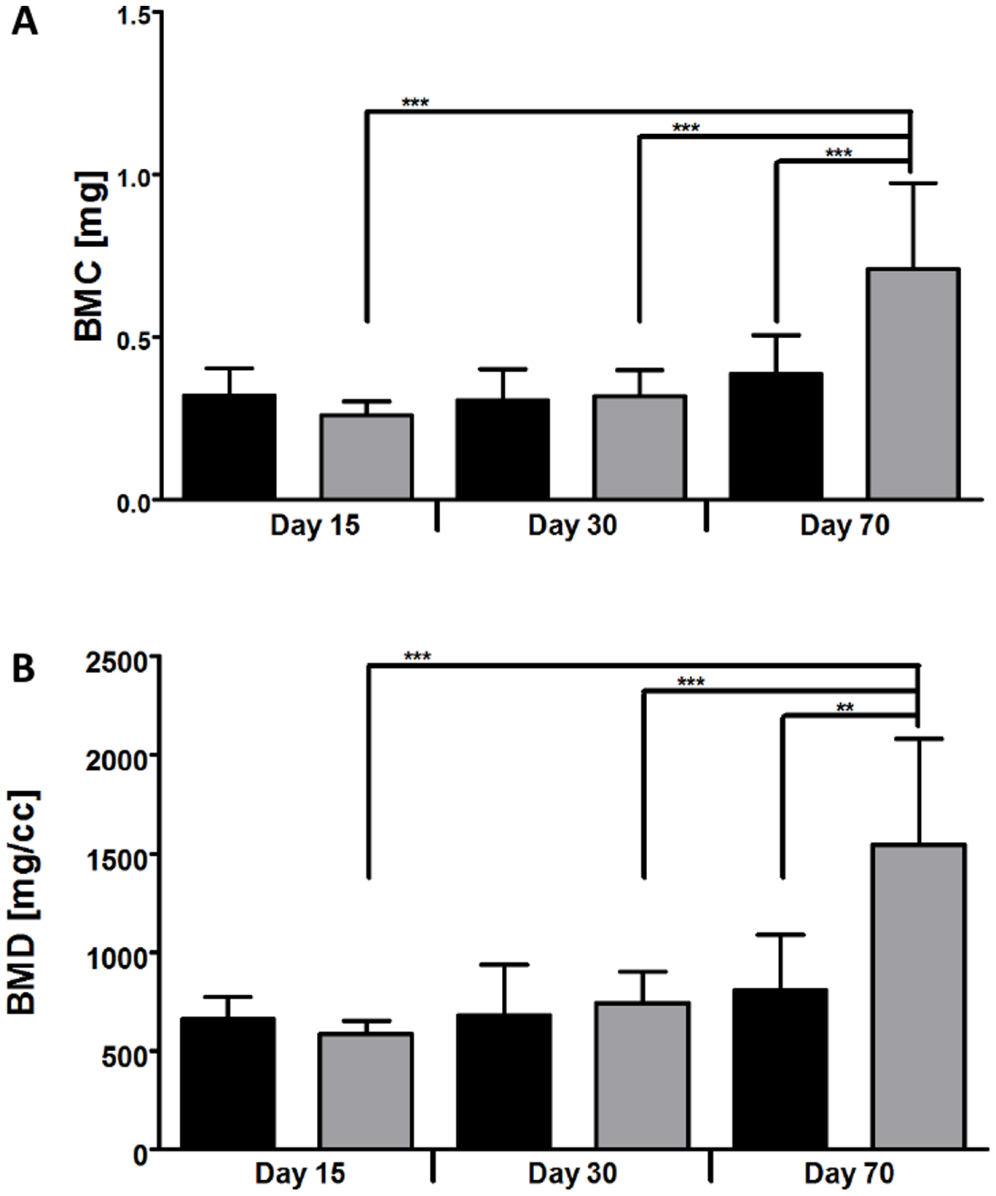
Ex *vivo* analysis of Bone mineral content (A) and Bone mineral density (B) by Micro-CT imaging. Regions of interest (ROI) were analysed in defects that were left empty (black columns) or received the beads (grey columns). Results are shown as means ± SD of 6 samples per condition. Significant differences are indicated with ** *p*≤0.01, and *** *p*≤0.001.

When evaluating Bone Mineral density (BMD) and Bone Mineral Content (BMC), there were no statistically significant differences between the empty defects and the defects that received the beads at day 15 and day 30. Values were strongly increased at day 70 for samples treated with beads in comparison to the empty defects (*p* ≤ 0.01) (Figure 3). When evaluating BMD and BMC for the samples that received beads, a significant increase was seen when comparing the two earlier with the latest time point.

**Figure 4:**
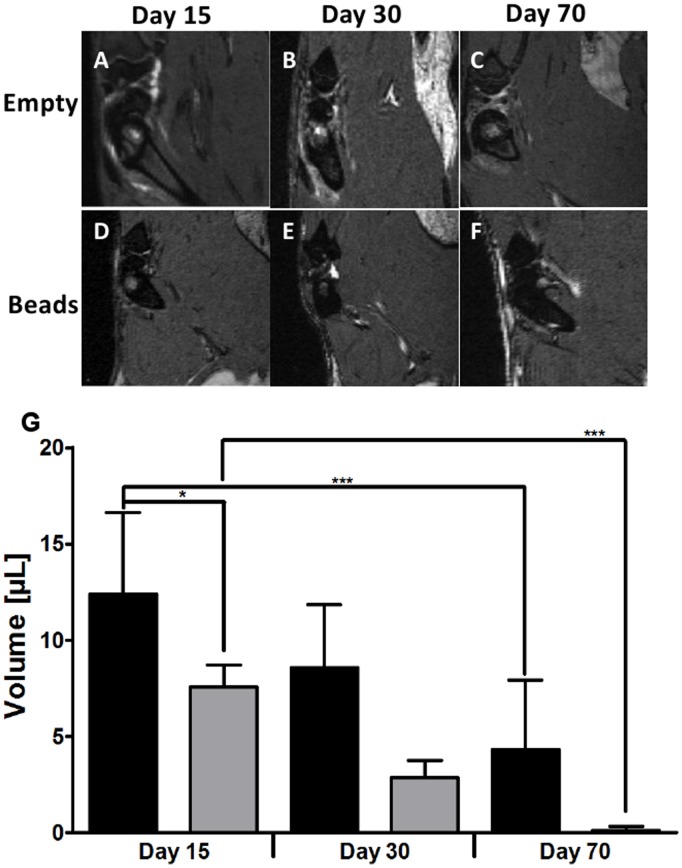
Representative images of MRI acquisition of condyles left empty (A–C) or after bead implantation (D–F) at 15 days (A, D), 30 days (B, E), and 70 days (C, F) after surgery. Quantification of the aqueous volume in µL of a fixed region of interest in defects that were left empty (black columns) or received the beads (grey columns), as obtained by longitudinal *in* *vivo* follow-up by Magnetic Resonance Imaging (G). Results are presented as means ± SD of 6 samples and significant differences are indicated with * *p*≤0.05, and *** *p*≤0.001.

**Figure 5:**
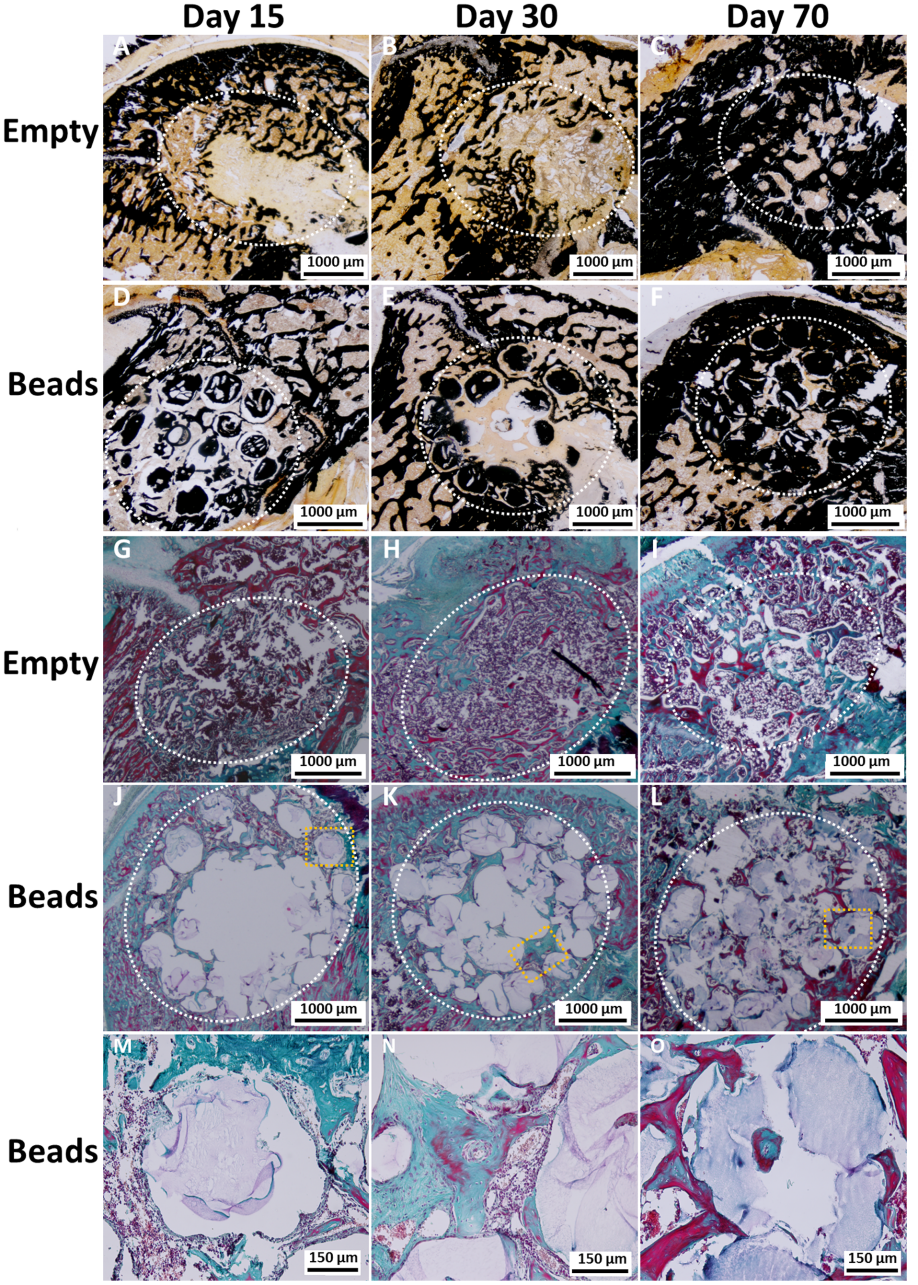
Representative images of histological staining of defects that were left empty (A–C, G–I) and for defects that received beads as implants (D–F, J–O). Calcified sections were stained with Von Kossa (A–F), while decalcified sections were stained with Masson’s Trichrome (G–O). Increase of mineralization is visible over time when comparing sections obtained at day 15 (A, D, G, J) with sections of day 30 (B, E, H, K) or those of day 70 (C, F, I, L). Areas of defects are marked with the dotted white line (A–L) and higher magnifications of the orange squares in J–L are shown in M–O.

Longitudinal *in* *vivo* follow-up by MRI did not reveal an inflammation in any of the conditions (Figure 4 A–F). The quantification of the aqueous volume showed a decrease for both conditions (Figure 4G). There were significant differences between the earliest and the latest time point for the two conditions (p≤ 0.001) and between the two conditions at day 15 (p≤ 0.05). Histological analysis did not reveal signs of inflammation at day 15 or at later time points (Figure 5). For defects that were left empty (Figure 5 A–C, G–I), cellularisation and mineralization started from the periphery of the defect. A similar process was observed with the beads, but cellular invasion also occurred within the defect (Figure 5 D–F, J–O).

**Figure 6:**
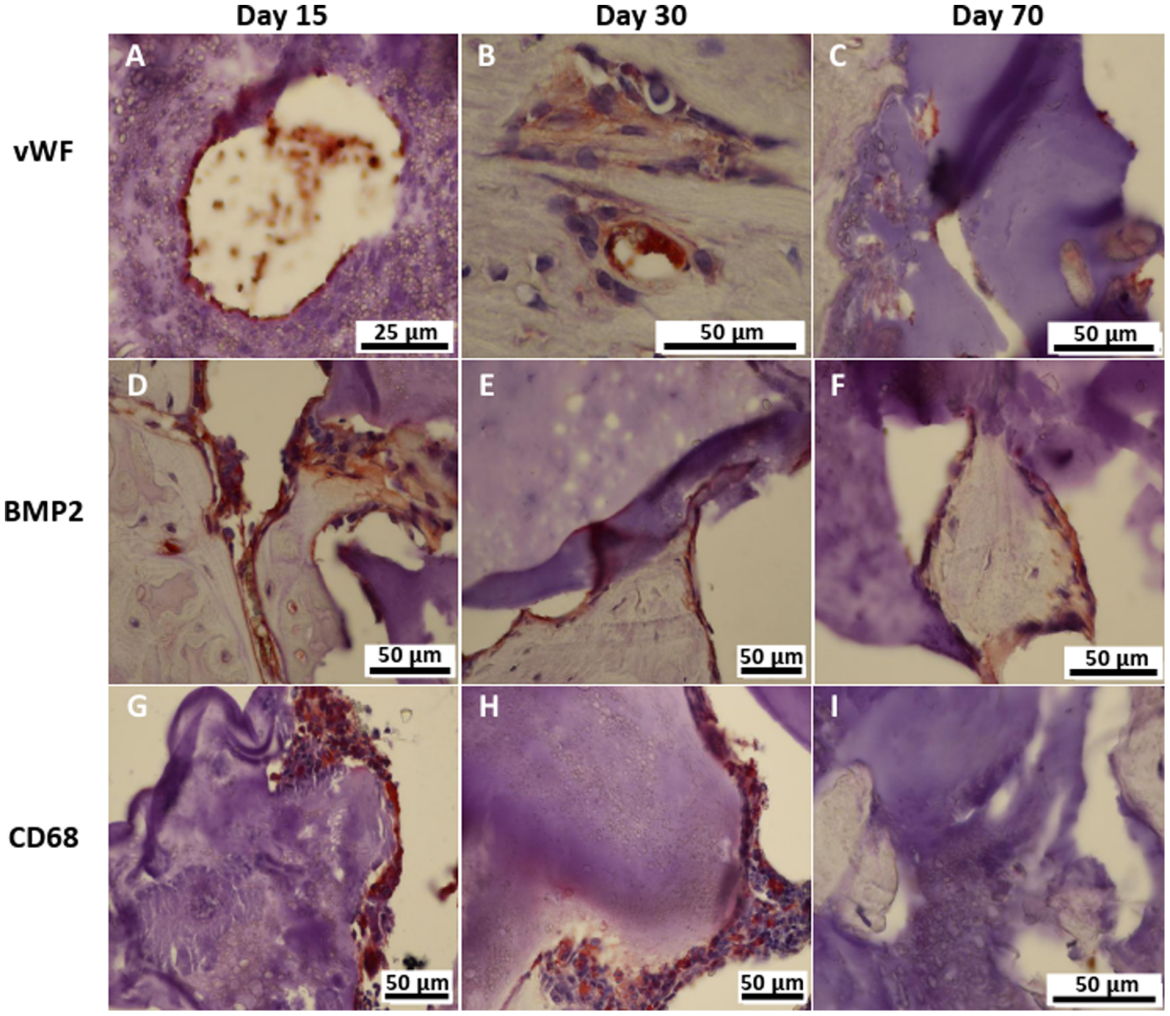
Representative images of immune-staining for cells in contact with the biomaterial using primary antibodies for von Willebrand Factor (A–C), Bone Morphogenetic Protein 2 (D–F) and CD 68 (G–I). Positive cells appear in brown and nuclei in dark blue.

Cellularisation of the material by endothelial cells forming vessels started within the first 15 days (Figure 6 A). Vessels were detected by vWF immune-staining in the trabecular-like structures (Figure 6 B) as well as within the material (Figure 6 A, C). BMP-2 positive cells secreting extracellular matrix were present at any time point, lining the trabecular-like structures, as well as in contact with the material (Figure 6 D–F). CD68 positive cells occurred mainly at the two earlier time points (Figure 6 G, H), while there was little presence of those cells at the latest time point (Figure 6 I). CD68 positive cells were seen in direct contact with the material.

**Figure 7:**
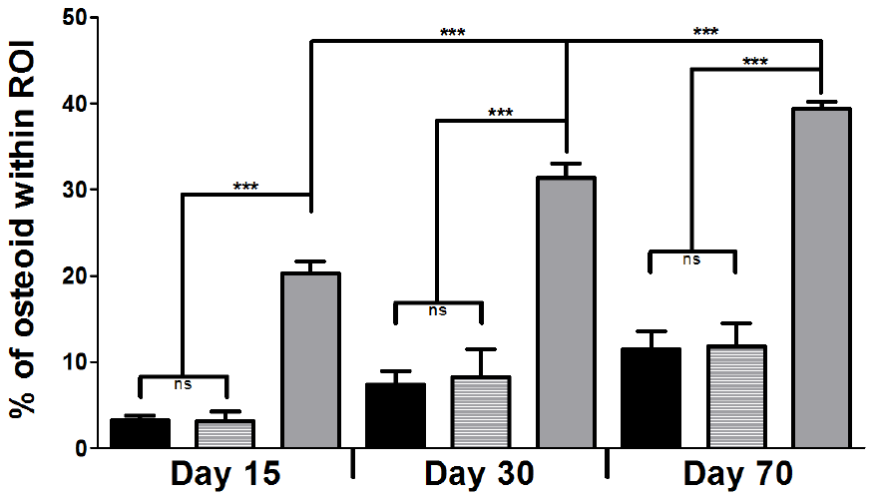
Histomorphometric analysis of newly formed osteoid in defects that were left empty (black columns) or received the beads (striped and grey columns). Osteoid surface over defect surface was expressed in percentage of osteoid within the region of interest (ROI), that was either the entire defect surface (black and striped columns) or the tissue surface around the beads (grey columns). Results are presented as means ± SD of 3 conditions and significant differences are indicated with *** *p*≤0.001.

Cellularisation was fully completed by day 70 for both conditions (Figure 5 I, L), but with different cellular structures. For the samples implanted with beads, cellular structures filled out all spaces between the beads with increasing interconnectivity and trabecular-like organization over time (Figure 5 D–F, J–L). Empty defects at day 15 after implantation showed few interconnected structures (Figure 5 A, G) and fibrosis that was still present at day 30 (Figure 5 H). At day 70, connective tissues were still visible, but most of the defect was filled with mineralized, trabecular structures (Figure 5 C, I). As it was seen in Von Kossa stained sections, the beads themselves, as well as the trabecular structures mineralized over time (Figure 5 D–F). Histomorphometric analysis of the newly formed tissue within the defect showed no significant difference at all time points between the two conditions when the percentage of osteoid surface was directly calculated within the surface of the entire defect (Figure 7). However, there was a significantly higher percentage of osteoid tissue formation at each time point when the surface of the beats was first subtracted from the defect surface in comparison to the empty defects (Figure 7). For the two calculations concerning the beads, there was a significant increase over time, while the increase in osteoid formation in empty defects was non-significant (not indicated in Figure 7).

## Discussion

In the present study, macroporous cross-linked beads based on a mixture of pullulan and dextran [49], [52] in association with nHA were used as a scaffold. This new scaffold, with injectable properties, was evaluated in a non-critical size bone defect, matching most clinical needs for small size defects.

Although most biomaterials are tested in critical-size defects, surgeons are often in need of materials to simply fill-up a small size defect that may not always be of a critical size [53]. The material tested in this experiment was previously implanted in the form of cylinders of 5 mm diameter and 6 mm depth in a critical size defect in rat femoral condyles and showed osteoconductive properties [36]. The goal of the present study was to investigate whether the material could modulate bone formation in a spontaneous bone regeneration model i.e. non-critical size defect to guide and activate bone mineralization and bone regeneration. The material was tested in the form of beads of 300–500 µm in diameter (Figure 1) to adapt to small size defect.

To fit specific needs for small size defects, the use of beads seems preferable. Scaffolds can be produced in any shape, but the advantage of small size beads is their capability to entirely fill up bone defects. The presented beads are sieved and can be produced at any desired diameter. In a previous study, the presented composite materials were used in the form of macroporous 3D cylinders [36], that showed limited access to invading cells and was therefore less cellularized than the material in the form of beads.

Although mineralization seemed to increase visually over time (Figure 2), it did not progress in the same way for the two conditions. It is known that defects left empty progress in bone repair from the periphery [54]. For that reason, the defects stayed rather empty in the median plan for the first 30 days (Figure 2 A, B). The presence of the beads seemed to allow a mineralization even within the defect; because trabecular-like structures were visible as early as 15 days after implantation (Figure 2 D). Thus, the material was used as a support for the invading cells, allowing a faster ingrowth of trabecular-like structures. Although those structures were less present within defects that were left empty, there was no significant difference for the BMC and the BMD in comparison with defects loaded with the material at day 15 and day 30. This is due to the fact that the entire defect was taken into account for the analysis and the defects left empty still repaired from the periphery. At day 70, bone repair was completed for the two conditions and BMC and BMD were significantly higher in the defect loaded with the beads. Overall, these results indicate that mineralization was delayed in empty defects in comparison with the condition that received the material. This indicates that beads of pullulan-dextran-nHA are suitable for a non-critical size defect and that mineralization was favored in the presence of the material.

As shown by histology, invading cells penetrated the scaffold within the first 15 days (Figure 5 J, M). Although pore sizes of the material were not investigated in this study, the beads allowed an attachment of invading cells, as cells, including vWF and BMP-2 positive cells, were visible in direct contact with the material at all time points (Figure 5 M–O, Figure 6). At day 30, Masson’s Trichrome stained sections (Figure 5 H) showed the presence of fibrous tissue that was not seen at any time point with the beads, allowing a better cellularisation of the defect. Similar results concerning tissue preservation and bone formation were observed with Bio-Oss^®^ collagen (Geistlich, Wolhausen, Switzerland) that contains demineralized bovine bone, considered as the gold standard biomaterial for alveolar sockets implantations to promote bone formation and ridge preservation [55]. In that study, non-critical-size defects in the extraction sockets of dogs were either filled with the material or left empty. Although the material did not enhance new bone formation, the profile of the ridge was better preserved with the graft.

As seen by immune-staining with a macrophage marker, CD68 positive cells were mainly seen at the two earlier time points (Figure 6 G, H). This is due to the cascade of events that is involved in inflammatory and wound healing responses following biomaterial implantation [56]. Directly after the implantation, polynuclear cells, monocytes and lymphocytes invade the defect site. The presence of a biomaterial as a foreign body provokes the infiltration with macrophages that differentiate and fuse to giant cells like osteoclasts at the end of the acute phase of the inflammation. At day 70, those processes are finished and CD68 positive cells are therefore no longer present.

MRI acquisition indicated a decrease of aqueous content over time for the two conditions (Figure 3 G), probably due to an increasing mineralization of the defect site over time, as seen by Micro-CT analysis. Although indicated in the graph, a direct comparison of the two conditions should be avoided, since the offset for the aqueous volume was not comparable for the two conditions. We did not measure the volume immediately after implantation because the animals were not transportable due to ethical reasons.

When analyzing Bone mineral density (BMD) and Bone mineral content (BMC), either the full defect or an area of interest, the distinction between newly formed bone and the biomaterial itself was difficult, since most micro-CT software choose thresholds representing the mineralized tissue. In the case of a biomaterial made of calcium phosphate containing hydroxyapatite, thresholding methods also capture the scaffold material in addition to the surrounding bone, as the densities of the bead material overlaps the heterogeneous densities found in bone and forming bone. We therefore decided to perform a histomorphometric analysis based on decalcified histological sections stained for osteoid tissue whose overall percentage was analyzed for the surface of the defect. While BMD and BMC values indicated an overall trend when using CaP based biomaterials, the histomorphometric analysis allows a more precise view on cellular processes. The main interest in performing this study was the evaluation of the increasing mineralized tissue surrounding the beads. Increasing osteoid formation, as analyzed by histomorphometric measurements, confirmed the increasing mineralization determined by Micro-CT analysis. As mentioned for the interpretation of the MRI results, a direct comparison of the two conditions seemed difficult, because the offset is not the same. Therefore, two series of measurements were performed. When the osteoid formation was expressed as percentage of the defect surface, there was no significant difference between the two conditions. But when the surface of the beads was first subtracted from the defect surface, the percentage of osteoid formation was significantly higher in samples that received beads (Figure 7). This result shows that beads have strong osteoconductive properties. The increasing presence of forming osteoid around the beads also indicates that the overall mineralization within the defect was not only due to the increased mineralization of the biomaterial itself as was shown before in an ectopic site [36].

While dextran is already used as a biomaterial in the form of hydrogels [57], [58] to deliver proteins and its synthetic derivatives can control the biological activity of growth factors [59], pullulan-based scaffolds have been evaluated more recently and studied as a support for smooth muscle cells [60]. Cylinders, disks or electrospun fibers of pullulan and dextran were evaluated *in* *vitro* as a composite matrix for endothelial progenitor cells [61], human dermal fibroblast [62], human bone marrow stromal cells [63], and human adipose derived stem cells [64]. The composition of the scaffold studied in the form of cylinders has shown abilities for mineralization and formation of a dense mineral collagenous tissue in both an ectopic site and a critical-sized orthotopic defect [36]. Here, we demonstrated the *in* *vivo* potential of pullulan-dextran-nHA beads for bone repair. It is known that materials based on polymers often need to be associated with cells or growth factors to achieve osteoconductive properties [65]. In this study, potentially injectable cell-free beads have been tested in a rat femoral medial-epicondyle defect of 3.5 mm diameter and 4 mm depth, and showed osteoconductive properties as shown by µCT, histology and histomorphometric analysis.

It is known that materials based on polymers often have inflammatory properties.

Regarding *in* *vivo* applications, the host response to the material is therefore an important parameter to look at [66], [67]. Polymers have been studied for their inflammatory properties and i.e. PLA or chitosan are long known to enhance the functions of inflammatory cells such as macrophages [68], [69], and recent studies focused on the evaluation of the influence of different acetylation degrees [70], as well as the 3D structure [71] on the inflammatory response to chitosan. Similar studies have been performed for hydroxyapatite, suggesting an influence of the material shape, size and sintering temperature on the inflammatory response [72]. The composite beads did not show an obvious inflammatory response, as investigated by MRI and histology (Figure 4 and 5), but CD68 positive cells were present as a consequence of the biomaterial implantation. A loss of aqueous volume for the two conditions was observed over time (Figure 4G). In case of a chronic inflammation, the aqueous loss would not decrease, but stay stable or even increase.

Although the present study demonstrated non-inflammatory and osteoconductive properties of the new injectable macroporous composite, improvements regarding its biodegradability, bone tissue invasion, and injectable properties are needed and ongoing. Since a cellularisation of the defect was visible and happened quite fast after implantation, it would be preferable to have beads of an injectable size that are resorbing over this time frame, to allow the replacement of the biomaterial itself by cells and extracellular matrix secreting cells. It is likely that the abundant vascularization observed inside the defect would allow a continuous infiltration of the site and a progressive replacement of the scaffold to achieve complete bone regeneration.

## Conclusion

Bone formation and mineralization for the defects filled with these beads were increased in comparison to empty defects. Cellular infiltration of the defect was well organized in trabecular-like structures, suggesting the use of beads for bone repair of small size defects. The beads are of an injectable size and can therefore be used to fill-up any type of bone defect of variable size and should become useful for surgeons in clinical applications.
